# Measurement of Creatine kinase and Aspartate aminotransferase in saliva of dogs: a pilot study

**DOI:** 10.1186/s12917-017-1080-x

**Published:** 2017-06-09

**Authors:** Asta Tvarijonaviciute, Tomas Barranco, Monica Rubio, Jose Maria Carrillo, Silvia Martinez-Subiela, Fernando Tecles, Juana Dolores Carrillo, José J. Cerón

**Affiliations:** 1 Animal Medicine and Surgery Department, School of Veterinary Medicine, University of Murcia, Campus of Espinardo s/n, 30100 Murcia, Spain; 2 Animal Medicine and Surgery Department, School of Veterinary Medicine, Universidad CEU Cardenal Herrera of Valencia, Valencia, Spain; 3 Garcia Cugat Foundation CEU-UCH Chair of Medicine and Regenerative 3 Surgery, Barcelona, Spain

**Keywords:** Aspartate aminotransferase (AST), Canine, Creatine kinase (CK), Muscular damage, Saliva, Serum

## Abstract

**Background:**

Muscle enzymes in saliva have been reported to be possible markers of heart and muscle damage in humans. The aim of this study was to assess if Creatine kinase (CK) and Aspartate aminotransferase (AST) activities could be measured in canine saliva, and to evaluate their possible changes in situations of muscle damage.

**Results:**

The spectrophotometric assays for CK and AST measurement in saliva of dogs showed intra- and inter-assay imprecision lower than 1 and 16% and coefficients of correlation close to 1 in linearity under dilution tests. Healthy dogs showed activities in saliva of CK between 27 and 121 U/L and AST between 46 and 144 U/L, whereas in saliva of dogs with muscle damage CK ranged between 132 and 3862 U/L and AST between 154 and 4340 U/L. Positive moderate correlations were found between saliva and serum activities of the two enzymes (CK, *r* = 0.579; *P* = 0.001; AST, *r* = 0.674; *P* = 0.001).

**Conclusions:**

CK and AST activities can be measured in canine saliva with commercially available spectrophotometric assays. In addition these enzymes show higher values in saliva of dogs with muscle damage and their values are moderately correlated with those of serum.

## Background

Saliva is a biological fluid that contains a certain number of elements common to other body fluids such as blood or urine in a sufficiently large consistency to take into interest their assays for biological analysis. Interest in saliva assays is increasing during last years [1, 2], with various scientific reviews indicating their advantages as a diagnostic medium such as its collection is safe, non-invasive, inexpensive, and simple. In addition it can be sampled repeatedly without discomfort to the patient [3].

Therefore saliva, especially in human medicine, has gained considerable attention as a possible alternate fluid to blood and urinary analysis [1] with a growing scientific literature describing the use of salivary biomarkers to determine diabetic and cardiac related risk factors, among others [4]. In dogs, analytes such as C-reactive protein [5] or cortisol [6, 7] have been measured in saliva and were found to be suitable biomarkers of inflammation and stress respectively.

Creatine kinase (CK) and Aspartate aminotransferase (AST) are two enzymes found in cytoplasm of muscle cells. They are considered to be markers of muscle cell damage, and an increase in these enzymes in serum may be an indicator of muscle stress or damage due to exercise [8–10] or muscle injuries [11, 12]. CK is primarily found in skeletal muscle, myocardium, brain and intestine [13]; although its main activity in serum is released from skeletal muscle [9]. While serum AST is less muscle specific than CK, since it is also present in liver and its activity in serum can increase due to liver damage [14]. In humans CK has been measured in saliva and it was indicated that plasma could be its main source [15].

CK and AST have been measured in serum of dogs as a marker of muscle damage under different conditions, such as hemilaminectomy or ovariohysterectomy [16], exercise [10], and after anesthesia [17]. However, to the authors’ knowledge no studies about CK and AST in saliva in dogs have been published previously. The knowledge about the possibility of measurement of CK and AST could be of both veterinarian and human interest, since the dog is a widely used experimental model for research.

The objective of the present study was to assess if CK and AST activities could be measured in canine saliva, and to evaluate their possible changes after processes that cause muscle damage. For this purpose, commercially available automated spectrophotometric CK and AST assays were validated in canine saliva and these two enzymes were measured in healthy dogs and in a group of dogs with increased serum CK and AST due to different causes.

## Methods

All experimental procedures were approved by the Local Ethical Committee of University of Murcia, and were performed in compliance with laws RD32/2007 and RD1201/2005 concerning animal experimentation in Spain.

### Assays

CK was measured by a commercial kit (Beckman Coulter, Brea, USA) based on the quantification of the NADPH formation derived from the action of CK on creatin phosphate as recommended by the International Federation of Clinical Chemistry (IFCC) [18]. AST was measured by a commercial kit (Beckman Coulter, Brea, USA) based on the quantification of the NADH consumption derived from the action of AST over aspartate and oxoglutarate as recommended by the IFCC [19]. Specimen volume used was 3 μl for CK and AST in the case of serum and 25 μl for CK and 3 μl for AST in case of saliva. A correction factor of [3/(3 + RV)]: [(25 + RV)/25] (where RV is reagent volume) was applied to the results of CK in saliva in order to take in consideration the different volume used. All the assays were performed in an automated biochemistry analyzer (Olympus AU600, Beckman Coulter, Brea, USA) at 37 °C. CK and AST assays showed an inter-assay imprecision and an inaccuracy of less than 5% in the daily quality control analysis done during the study. Manufacturer’s control solutions of two different values were used for the quality control analysis (Beckman-coulter, Lot 0037 and 0038).

### Analytical validation

For analytical validation of both methods the following parameters were calculated.

#### Precision

The intra-assay coefficient of variation (CV) was calculated after analysis of 2 saliva specimens with different CK and AST concentration 5 times in a single assay run. The inter-assay CV was determined by analyzing the same specimens in 5 separate runs, carried out on different days, being the specimens stored at −80 °C.

#### Accuracy

It was evaluated indirectly by linearity under dilution. For this purpose, two canine saliva specimens were serially diluted with bidistilated water.

#### Limit of detection

This was calculated on the basis of data from 10 replicate determinations of the zero standard (bidistilated water) as the mean value plus 3 standard deviations.

### Animals

A total of 27 dogs were included in the present study. Thirteen of these animals were healthy dogs belonging to staff and students of Murcia University and were used as controls. None of the dogs presented abnormalities at physical examination, or in the CBC and biochemical profile, and did not have evidence of periodontal disease. In particular, serum activities of AST and CK were lower than 50 UI/L and 250 U/L respectively, values which represent the higher limit of the reference interval of our laboratory.

All animal were adults with a mean (range) age of 5.1 (2.0-8.0) years and a median (range) body weight (BW) of 19.3 (7.0-29.0) kg. Six dogs were mongrels, three were Beagles, two were Labrador Retrievers, one was Golden Retriever and one was Poodle.

In order to evaluate if CK and AST increased in saliva in dogs with muscle damage, fourteen dogs with increased serum CK and AST due to diverse causes such as traumas or surgery were included in the study. The saliva and blood specimens were taken at time of admission. In this group of dogs age ranged between 0.5 and 10.0 years (median 6.4 years), and BW was between 1.5 and 40.0 kg (median 25.5 kg). Seven dogs were mongrels, three were Yorkshire terriers and there was one dog of each of the following breeds: San Bernardo, Boxer, German Shepherd and Spanish water dog. In all cases, the exclusion criteria were: the saliva specimens obtained had not enough volume for measurements; and presence of periodontal disease since salivary CK and AST was shown to increase in humans with periodontal disease [20].

### Saliva and blood sampling

Saliva specimens were obtained by placing a sponge in dog’s mouth for 1–2 min as previously described [5, 21]. Then the sponge was placed into the Salivette (Salivette®, Sarstedt AG &Co., Nümbrecht, Germany) device for centrifugation (P Selecta®, JP Selecta S.A, Barcelona, Spain) at 3000 x g 10 min [5, 22]. After saliva collection, venous blood specimens (2 mL) were collected from the jugular vein into plain tubes (Vacutainer®, Plymouth, United Kingdom). Tubes were let to clot at room temperature for 30 min and centrifuged (2000 x g, 10 min) for careful removal of the serum.

The saliva and blood specimens were processed and measured in less than 1 h after collection, and serum and centrifuged saliva samples were keep at 4 °C until were measured.

### Statistical analysis

Normality of the data distribution was evaluated with a Kolmogorov-Smirnov test and, since data was not normally distributed, non-parametric tests were used. Differences in serum and salivary CK and AST between healthy controls and dogs muscle enzymes in serum out of reference interval were evaluated using the Mann–Whitney test. Correlations between serum and saliva were calculated using the Spearman correlation coefficient. The level of significance was set at *P* < 0.05. Statistical analyses were performed with computer software (Graph Pad Prism Version 7 for Windows, Graph Pad software, La Jolla, CA).

## Results

**Fig. 1:**
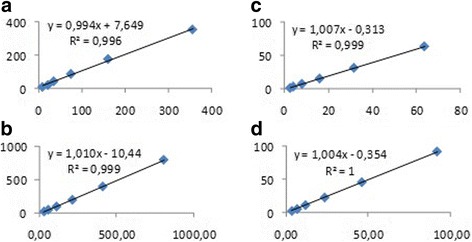
Linearity under dilution of two saliva specimens with different CK and AST concentrations. CK (**a**,**b**) and AST (**c**,**d**) concentrations

CK in saliva showed intra- and inter-assay imprecision less than 1 and 16%, respectively, while AST in saliva showed intra- and inter-assay imprecision less than 1 and 7%, respectively (Table 1). Linearity under dilution resulted in coefficient of correlation close to 1 in both cases (Fig. 1). Limit of detection for salivary CK was 6.01 U/L (mean, 2.05; SD, 1.32) and for salivary AST was 2.11 U/L (mean, 0.82; SD, 0.43).

**Table 1:** Intra- and interassay coefficients of variation (CV)

Assay	Comparison	Specimen	mean	Sd	CV (%)
CK, U/L	Intra	Specimen 1	64.6	0.12	0.2
		Specimen 2	293.5	1.46	0.5
	Inter	Specimen 1	49.1	7.26	14.8
		Specimen 2	182.8	27.74	15.2
AST, U/L	Intra	Specimen 1	91.8	0.36	0.4
		Specimen 2	639.3	3.19	0.5
	Inter	Specimen 1	84.7	5.75	6.8
		Specimen 2	619.1	15.47	2.5

**Fig. 2:**
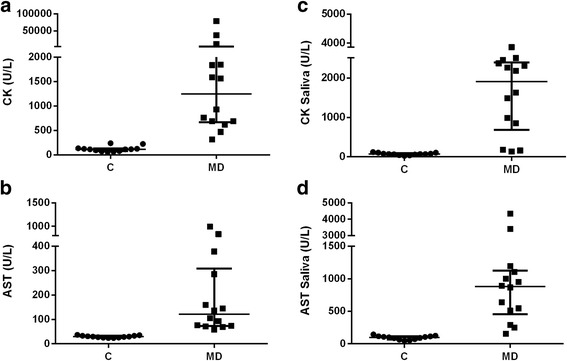
Serum and saliva CK and AST in healthy dogs and dogs with muscle damage. Serum and saliva CK (**a** and **b**, respectively) and AST (**c** and **d**, respectively) in controls (C, *n* = 13) and dogs with muscle damage (MD, *n* = 14)

Serum CK activity in healthy controls ranged between 57.9 and 239 U/L, whereas it was significantly higher in dogs with muscle damage (*P* < 0.001) with values between 319 and 78,650 U/L (Fig. 2a). Serum AST in healthy controls ranged between 23.8 and 36.9 U/L and dogs with muscle damage showed significantly higher activity in serum AST (*P* < 0.001) with values between 58.3 and 988 U/L (Fig. 2b).

Salivary CK in healthy controls ranged between 27 and 121 U/L, whereas dogs with muscle damage showed a significantly higher activity in saliva CK (*P* < 0.001) with values between 132 and 3862 U/L (Fig. 2c). AST in saliva in healthy controls ranged between 46.8 and 144 U/L, whereas dogs with muscle damage showed a significantly higher activity in saliva AST (*P* < 0.001) with values between 154 and 4340 U/L (Fig. 2d).

When data from healthy controls and those with muscle damage were pooled, positive correlations were observed between serum and salivary CK (*r* = 0.579; *P* = 0.001) and between serum and salivary AST (*r* = 0.674; *P* < 0.001).

## Discussion

Serum activities of CK and AST reflect damage of muscle and are useful markers to determine the condition of muscle tissue both in dogs [10, 13, 23, 24] and humans [25, 26]. In particular, increases in CK following physical activity has been associated with muscle damage in both racing and hunting dogs [9] and similar findings have been described in humans [8, 25].

The assays used in our study showed an adequate intra-assay imprecision and accuracy to measure the activity of both enzymes in saliva. Therefore CK and AST can be measured in saliva in dogs with automated spectrophotometric assays available for routine use in clinical pathology laboratories. The inter-assay imprecision was higher than 15% for CK, that is considered as the acceptable limit [27]; this fact could be related with the stability of the enzyme under storage and further studies should be made to elucidate the reason. In case of using assays from a different manufacturer than those used in our study, an optimization of specimen volume and analytical validation of the assay would be recommended.

Our study reports for the first time the existence of CK and AST activities in saliva of dogs. In healthy dogs of our study CK showed lower values in saliva compared to serum which agrees with previous reports made in humans [15]. The lower values of CK in saliva could be due to the existence of a limit in the transfer of CK from plasma to saliva as it has been suggested in human for CK and other analytes such as NT-ProBNP [15, 28]. This situation seems not occur in the case of AST, that showed higher values in saliva than in serum. Additional studies should be performed to elucidate the reasons of the higher values of AST in saliva compared with serum in dogs.

Dogs with muscle damage showed significant increases in CK and AST in saliva and there was no overlap in the values of these enzymes between healthy dogs and dogs with muscle damage. In dogs with muscle damage the values of CK in saliva did not reach the values in serum (upper value in saliva of 3862 versus 78,650 U/L in serum) and again a limit in the transfer of CK from plasma to serum could be suggested to explain this fact. On the other hand in the case of AST values in saliva of dogs with muscle damage were higher than in serum (upper value in saliva of 4340 versus 988 U/L in serum), and further studies should be undertaken to elucidate the mechanisms that can produce this effect.

Significant correlations were obtained between salivary and serum activities of CK and AST in dogs and this would indicate a possible pass of the enzymes from serum to saliva. However these correlations were moderate, similarly to what has been found in humans [15]. One cause of this moderate correlation could be due to the different dynamics after a muscle damage that the enzymes can have in serum and saliva. In addition, from the analytical point of view it should be considered the different kinetics that the enzymatic reactions had in serum and saliva, although the sample dilution and further dilution in the reagents would likely minimize the matrix effect. Further studies about the process allowing muscle enzymes to filter from plasma to saliva, as well factors that could increase or decrease this transfer, would be recommended. In addition the possibility of enzyme synthesis by the salivary glands should be explored. This would allow a better understanding of the clinical significance of muscle enzymes in saliva and their correlation with serum.

Saliva analysis could be a potential suitable alternative to serum due to its non-invasive collection, which could be convenient in cases where dogs are reluctant to blood sampling or in young dogs of small breeds. However, it is important to point out some limitations that the measurements of CK and AST in saliva could have from a practical point of view. The first one is that in some cases it can not be obtained enough volume for analysis, as it have been previously described for salivary cortisol measurements in dogs [29]. The second one is that the presence of gingivitis and/or dental plaque is known to produce increases in salivary CK and AST in humans [20], and therefore the interpretation of these enzymes in saliva of dogs with these diseases should be made with caution.

This is a pilot study and further studies with a larger population of dogs should be made to confirm these findings. In addition although in our study the specimens have been analyzed in the first hour after collection, it would be of interest to evaluate the influence of different storage conditions in the stability of these enzymes in saliva. Furthermore, additional studies could be of interest to evaluate the behavior of the different CK isoenzymes in saliva and also the dynamics of CK and AST in serum and saliva after an experimental induced muscle damage as well in dogs with and without periodontal disease and gingivitis.

## Conclusions

Results of the present study indicate that CK and AST activities can be measured in canine saliva with commercially available spectrophotometric assays. In addition these enzymes show higher values in saliva of dogs with muscle damage compared with healthy dogs and their values have a moderate correlation with those of serum.
